# Comparison of the analytical and clinical performances of two different routine testing protocols for antinuclear antibody screening

**DOI:** 10.1002/jcla.23914

**Published:** 2021-08-04

**Authors:** Concepción González Rodríguez, Sandra Fuentes Cantero, Antonio Pérez Pérez, Francisco Javier Vázquez Barbero, Antonio León Justel

**Affiliations:** ^1^ Department of Biochemistry Hospital Universitario Virgen Macarena Seville Spain

**Keywords:** antinuclear antibodies, diagnosis, EliA, enzyme‐linked immunosorbent assay, Indirect immunofluorescence, method comparison, systemic autoimmune rheumatic diseases

## Abstract

**Background:**

The diagnosis of systemic autoimmune rheumatic diseases (SARD) is based on the detection of serum antinuclear antibodies (ANA) for which indirect immunofluorescence (IIF) is the golden standard. New solid‐phase immunoassays have been developed to be used alone or in combination with the detection of extractable antinuclear antibodies (ENA) to improve SARD diagnosis. The purpose of this study was to compare the clinical performances of different ANA screening methods alone or in combination with ENA screening methods for SARD diagnosis.

**Methods:**

A total of 323 patients were screened for ANA by IIF, EliA™ CTD Screen, and ELISA methods. Agreements were calculated between the methods. Then, EliA™ CTD Screen positive samples were screened for ENA by line immunoassay (LIA) and fluorescence enzyme immunoassay (FEIA).

**Results:**

The diagnostic accuracy of EliA™ CTD Screen (79% sensitivity and 91% specificity) was better than that of ELISA or IIF. The combination of EliA™ CTD plus IIF had the highest sensitivity (93%). ENA determination revealed that Ro52 and Ro60 were the most prevalent specificities. The use of IIF alone was not able of detecting up to 36% of samples positive for Ro52, and 41% for Ro60.

**Conclusions:**

EliA™ CTD Screen has a better diagnostic performance when compared to IIF and ELISA. The combined use of EliA™ CTD Screen and IIF clearly improves the rate and accuracy of SARD diagnosis. The use of EliA™ CTD Screen as first‐line screening technique allows the detection of antibodies, which could not be detected by IIF alone.

## Graphical abstract

There is still room for improvement in ANA screening testing. Currently there are different methods available for systemic autoimmune rheumathic disease sift, but the optimization of screening protocols does not only rely on the selection of the best method–there is no perfect test‐ but in the detection of the optimal combination for the specific laboratory routine. In our study we compared the performance of two protocols in our routine samples: solid phase test+IIF and ELISA+IIF. IIF together with EliA CTD Screen (solid phase test) resulted in the highest sensitivity, while EliA CTD Screen showed the best sensitivity/specificity balance.

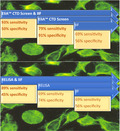

## 1 INTRODUCTION

Systemic autoimmune rheumatic diseases (SARD), also known as connective tissue diseases (CTD), including all diseases triggered by the formation of immune complexes that enter the circulation, are then deposited in different tissues and organs, and cause damage.^1^ The detection of antinuclear antibodies (ANA) and of antibodies to extractable nuclear antigens (ENA) is used in the diagnosis of SARD and in the prediction of an early onset of disease.^2^

Indirect immunofluorescence (IIF) on HEp‐2 cells (human epidermoid laryngeal carcinoma cells) has been defined as the reference screening method for ANA in the clinical laboratory routine.^3^ However, IIF is a labor‐intensive, time‐consuming procedure and has poor reproducibility due to the subjective interpretation of results.^4^ Therefore, in 2014, an international workgroup of experts representing 15 European countries developed a set of recommendations for the appropriate assessment and interpretation of ANA detected by different methods. According to this expert panel, alternative assays might be preferred for ANA screening when clinical suspicion is strong; and when the results of these alternative methods are negative, IIF should be used for the definitive diagnosis.^3^ In this regard, various automated solid‐phase immunoassays (such as enzyme‐linked immunosorbent assay (ELISA) and fluorescence enzyme immunoassay‐based (FEIA) assays) have been developed to be used as first‐line screening methods in SARD diagnosis.^2^ ELISA is a plate‐based assay technique in which antigens of HEp‐2 cell extracts are immobilized on a solid surface, whereas FEIA is designed as a sandwich assay where a mix of antigens is coated to the solid phase. Antinuclear autoantibodies bind to these antigens, which are coupled to an enzyme‐linked antibody, producing a fluorescent signal upon binding.^2^

Such systems are attractive alternatives to IIF, not only because of the automated process but also because of the improved specificity compared to IIF. Moreover, it has been widely demonstrated that a combination of these two techniques may improve the diagnostic accuracy of ANA screening.^5^, ^6^, ^7^, ^8^

In routine laboratory testing, after a positive result is obtained on a screening platform, it is useful to determine the specificity of the antibody using different ENA testing platforms due to their prognostic and diagnostic power. Taking into account, the availability of different methods for SARD diagnosis, it is important to evaluate the performance of different tests in order to determine which ANA and ENA combination performs best and is more sustainable.

The aim of this study was the comparative analysis of three different ANA screening methods (EliA™ CTD Screen, IIF, and ELISA). An analysis was carried out considering single or combination tests (EliA™ CTD Screen plus IIF vs. ELISA plus IIF). We also evaluated the potential ANA plus ENA combination testing in SARD diagnosis using two different ENA screening methods (LIA and FEIA).

## 2 MATERIALS AND METHODS

### 2.1 Patients

Cross‐sectional study including samples referred for ANA testing from April to August 2019. A total of 323 patients from the primary care, rheumatology, nephrology, and internal medicine services of the Virgen Macarena University Hospital (Seville, Spain) were evaluated. The samples were collected randomly, including just one sample per patient. The samples were retrospectively classified, according to patients final clinical diagnosis, into the following groups: SARD group, *N* = 147 (including systemic lupus erythematosus, Sjögren's syndrome, mixed connective tissue disease, polymyositis/dermatomyositis, systemic sclerosis, undifferentiated connective tissue diseases, rheumatoid arthritis, and vasculitis); Organ‐specific autoimmune disease group, *N* = 31; Malignancies group, *N* = 12 and Non‐autoimmune diseases, *N* = 147). This study was approved by the local Ethics Committee of the Virgen Macarena University Hospital.

### 2.2 Antinuclear antibodies (ANA) Screening

ANA screening was performed with the following three techniques:

*IIF*: ANA was performed using HEp‐2^®^ cells (Immunoconcepts) (screening dilution 1:80) using ≥1:80 cut‐off which allows the detection of antibodies against a wide variety of nuclear molecules and antigens located in the cytoplasm, including those in mitotic cells. ANA IIF were carried out by Dr. González and Dr. Pérez, who have 30 and 7 years of experience, respectively; using an automated microscope (Image Navigator, Nikon) and SIExpert software (Palex Medical, Sant Cugat del Vallés).

*FEIA*: The EliA™ CTD Screen (Thermo Fisher Scientific) was performed using the Phadia 250 instrument. This assay contains a mixture of the following antigens: dsDNA, Ro52, Ro60, SSB, Sm, U1RNP (RNP‐70, A, C), Jo‐1, Scl‐70, CENP‐B, Mi‐2, RNA Pol III, PM‐Scl, PCNA, Ribosomal P, and Fibrillarin. The ratios of test sample to calibrator (cut‐off) recommended by the manufacturer were used: <0.7, negative; 0.7–1, equivocal; >1, positive.

*ELISA*: RELISA (Immunoconcepts) a qualitative indirect enzyme immunoassay was used. Stabilized antigens (dsDNA, histones, SSA/Ro60, SSB/la, Sm, Sm/RNP, Scl70, Centromere, Jo‐1, PCNA, Ribosomal P, Mi‐2 mitochondrial) and other antigens from HEp‐2 nucleus and cytoplasm are coated onto the surface of the microwells to serve as antigenic substrates. Cut‐off values recommended by the manufacturer were used: <10 U/ml, negative; 10–15 U/ml, borderline; >15 U/ml, positive.

For combined testing (ie, when more than one of these tests were used), patients with a positive result in one of the tests were considered positive, and patients with negative results in both tests were considered negative.

### 2.3 Extractable nuclear antigens (ENA) Screening

EliA^™^ CTD Screen positive samples (*n* = 123) were analyzed for the following ENA specificities using two different methods:

*LIA*: Euroline (Euroimmun), including RNP‐U 1(70 kDa+A+C), Sm, SSA/Ro60, SSA/Ro52, SSB, Scl70, Jo‐1, CENP‐B, PCNA, Histones, Ribosomal P proteins, PM‐Scl, Mitochondrial, and Nucleosomes. The intensity of the resulting staining is proportional to the antibody concentration in the sample. Therefore, semi‐quantitative cut‐off values recommended by the manufacturer were used as follows: negative (0–10 intensity of the resulting staining); weak positive (11–25); moderate positive (26–50), and strong positive (>50).

*FEIA*: EliA specificities (Thermo Fisher Scientific), including SmDP, Rib P, PCNA, U1RNP, Ro, Ro52, Ro60, La, CENP, Scl‐70s, Fibrillarin, RNA Pol III, PM‐Scl, Jo‐1, and Mi‐1. Cut‐off values recommended by the manufacturer were used: ≤1.0 U/ml, negative; >1 U/ml, positive.

### 2.4 Statistical analysis

The differences between the results obtained from different methods were analyzed by chi‐square, phi coefficient, and contingency coefficient tests. Cohen's kappa coefficients were used to estimate the measuring agreement among methods. Statistical analyses were performed using the software SPSS Statistics v25.

## 3 RESULTS

### 3.1 Demographics

In this patient cohort, the prevalence of SARD was 41.2% (116 of 246 women, and 17 of 77 men). The median age of the patients was 56 ± 16 years. Table 1 describes the demographic data for each group.

**TABLE 1:** Demographics and patients’ clinical profile description

		*N*	%	Ratio W/M	Age (average)
Non‐autoimmune disease (*n* = 147)				2/1	59
Arthrosis		23	7.12		
Arthralgias		16	4.95
Neuropathies		11	3.40
Psoriasis		6	1.86
Fibromyalgia		17	5.26
Nephropathies		25	7.74
Skin lesions		7	2.17
Thrombopenia		11	3.40
Synovitis		5	1.55
Raynaud's and vascular disorders		7	2.17
Pulmonary thromboembolism		3	0.92
Infections		2	0.62
Others		14	4.33
Malignancies (*n* = 12)				3/1	62
Malignancies		12	3.71		
Organ‐specific autoimmune diseases (*n* = 31)				3.3/1	56
Autoimmune thyroid diseases		8	2.48		
Autoimmune liver diseases and cholangitis		11	3.40
Diabetes		1	0.31
Other arthritis (undifferentiated, psoriasis)		7	2.17
Crohn´s disease		2	0.31
Celiac disease		2	0.62
Myasthenia gravis		1	0.31
SARD (*n* = 133)				8/1	54
Rheumatoid arthritis		22	6.81		
Mixed connective tissue disease		5	1.55
Systemic sclerosis		11	3.40
Systemic lupus erythematosus		38	11.76
Dermatomyositis/polymyositis		4	1.24
Antiphospholipid syndrome			
Primary		4	1.24
Secondary		4*	1.24*
Sjögren syndrome		39	12.07
Undifferentiated connective tissue diseases		6	1.86
Systemic vasculitis			
ANCA+		1	1.24
ANCA−		3	
TOTAL		323	100%

Abbreviations: M, men; W,women.

### 3.2 Comparison of ANA Screening platforms

The results of ANA screening with IIF, ELISA, and EliA™ CTD Screen were obtained and compared. As shown in Table 2 183 of the results were consistent (56.7%) and 140 results (43.3%) were contradictory between IIF and EliA™ CTD Screen techniques, whereas 251 of the results were consistent (77.7%) and 72 results (22.3%) were contradictory between ELISA Screen and EliA™ CTD Screen techniques.

**TABLE 2:** Comparison of ANA screening results by EliA™ CTD, IIF and ELISA screen

	EliA™CTD SCREEN		Total
	Negative	Positive	
IIF			
Negative	104 (32.2%)	44 (13.6%)	148 (45.8%)
Positive	96 (29.7%)	79 (24.4%)	175 (54.2%)
Total	200 (61.9%)	123 (38%)	323 (100%)
ELISA Screen			
Negative	155 (48%)	27 (8.3%)	182 (56.3%)
Positive	45 (13.9%)	96 (29.7%)	141 (43.6%)
Total	200 (61.9%)	123 (38%)	323 (100%)

Bivariate analysis between EliA™ CTD Screen, IIF, and ELISA techniques measured by Pearson's chi‐square test revealed statistically significant differences between each test (*p *< 0.05) (Table 3). Phi coefficient and contingency coefficient were also calculated and presented in Table 3. Method agreement analysis (Kappa coefficient) revealed that the agreement between EliA™ CTD Screen and ELISA (moderate, 0.540) was higher than the agreement between EliA™ CTD and IIF (weak, 0.150) or between ELISA and IIF (weak, 0.203). Importantly, the agreement between combined tests (EliA™ CTD plus IIF vs ELISA plus IIF) was stronger (strong, 0.814). Discrepant results are shown in Table4.

**TABLE 3:** Comparative statistical tests and agreement coefficients between different ANA screening platforms alone or combined

	Pearson's chi‐square test	Phi Coefficient Φ	Contingency Coefficient	Kappa Coefficient
EliA™ CTD vs IIF	8.079 (*p* < 0.004)	0.158	0.156	0.150
EliA™ CTD vs ELISA	95.54 (*p* < 0.0001)	0.544	0.478	0.540
ELISA vs IIF	13.98 (*p* < 0.0001)	0.208	0.204	0.203
EliA™ CTD& IIF vs ELISA & IIF	214.1 (*p* < 0.0001)	0.814	0.631	0.814

Abbreviations: IIF, indirect immunofluorescence.

**TABLE 4:** (A) Discrepant results for the three methods compared. (B) Description of titer and pattern for discrepant results between IIF and EliA or ELISA

(A)						
	EliA +ELISA‐	ELIA ‐ELISA+	IIF‐EliA+	IIF‐ELISA+	IIF+EliA‐	IIF+ELISA‐
*N*	27	45	44	48	96	82

(B)						
	IIF+EliA‐			IIF+ELISA‐		
IIF Pattern	29.0% Nuclear Homogeneous (AC−1)			29.3% Nuclear Homogeneous (AC−1)		
	29.0% Nuclear Fine Speckled (AC−4)			30.5% Nuclear Fine Speckled (AC−4)		
	8.3% Nuclear Dense Fine Speckled (AC−2)			9,8% Nuclear Dense Fine Speckled (AC−2)		
	2.0% Nuclear Coarse Speckled (AC−5)			2.4% Nuclear Coarse Speckled (AC−5)		
	12.5% Nucleolar (AC−8,9,10)			9.8% Nucleolar (AC−8,9,10)		
	5,2% Reticular/AMA (AC−21)			6.0% Reticular/AMA (AC−21)		
	4.0% Discrete nuclear dots (AC−6,7)			3.6% Discrete nuclear dots (AC−6,7)		
	2.0% Centromere (AC−3)			1.2% Centromere (AC−3)		
	2.0% Fibrillar Cytoplasmic (AC−15,16,17)			2.4% Fibrillar Cytoplasmic (AC−15,16,17)		
	3.0% Cytoplasmic Speckled (AC−18, 19, 20)			3.6% Cytoplasmic Speckled (AC−18, 19, 20)		
	2.0% Other			24.4% Other		
IIF titer	1/80: 66.7%			1/80: 70.7%		
	1/160: 20.8%			1/160: 17.1%		
	1/320; 10.4%			1/320: 11.0%		
	>1/640: 2.1% a			>1/640: 1.2%		

Next, SARD diagnosis performances of three screening platforms were compared (Table 5). For EliA^™^ CTD Screen, sensitivity was 79% (95% CI: 72%–86%), specificity was 91% (95% CI: 86–95%), and positive and negative likelihood ratios (LR) values were 8.33 (2.05–3.50) and 0.23 (0.32–0.55), respectively. IIF had the following values: sensitivity 69% (95% CI: 61%‐77%), specificity 56% (95% CI: 49%‐63%), positive LR 1.58 (1.30–1.93), and negative LR 0.55 (0.41–0.73); while ELISA showed a sensitivity of 69% (95% CI: 61%–77%), a specificity of 74% (95% CI: 68%‐80%), positive LR 2.68 (2.05–3.50), and negative LR 0.42 (0.32–0.55). Therefore, it can be concluded that EliA^™^ CTD Screen had higher sensitivity, specificity, and positive LR, and better negative LR compared to both IIF and ELISA.

**TABLE 5:** Diagnostic performances ANA screen platforms (alone or combined)

	EliA^™^ CTD	IIF	ELISA	EliA™ CTD +IIF	ELISA +IIF
Sensitivity (%)	79%	69%	69%	93%	89%
Specificity (%)	91%	56%	74%	50%	45%
LR+	8.33	1.58	2.68	1.86	1.63
LR−	0.23	0.55	0.42	0.14	0.23

Abbreviations: LR, Likelihood ratio; IIF, indirect immunofluorescence.

Moreover, when the performance of EliA™ CTD Screen plus IIF was compared to ELISA plus IIF screening, EliA™ CTD Screen plus IIF screening showed higher sensitivity (93% vs. 89%) and specificity (50% vs 45%), and higher positive LR (1.86 vs. 1.63) and lower negative LR (0.14 vs. 0.23) values.

### 3.3 Specific ENA profile

The antigenic specificities were assessed using two methods: EliA™ and LIA. Both platforms displayed the highest positivity rates for Ro60 and Ro52 antibodies that are most commonly reported in the literature for SARD diagnosis. Therefore, the percentage of these antigens (as calculated from the total positive specificities of each method) was assessed in systemic autoimmune diseases (Table 6). For the two most prevalent systemic autoimmune diseases, systemic lupus erythematosus, and Sjögren's syndrome, the following positivity percentages were measured: Systemic Lupus Erythematosus, EliA™‐Ro52 = 3.42% and Ro60 = 8.12%, LIA ‐ Ro52 = 6.59% and Ro60 = 9.89%; and Sjögren's Syndrome, EliA™‐Ro52 = 8.55% and Ro60 = 10.26%, LIA‐Ro52 = 12.64%, and Ro60 = 11.5%.

**TABLE 6:** Frequency of Ro52 and Ro60 antibodies per autoimmune disease assessed by EliA™ and LIA

	EliA™ CTD		LIA	
	Ro52	Ro60	Ro52	Ro60
Systemic lupus erythematosus	8 (3.42%)	19 (8.12%)	12 (6.59%)	18 (9.89%)
Sjögren's syndrome	20 (8.55%)	24 (10.26%)	23(12.64%)	21 (11.53%)
Systemic sclerosis	1	1	1	3
MCTD	1	0	3	0
DM‐PM	2	1	1	1
Undifferentiated CTD	1	1	1	1
Other	5	9	8	8
Total	38	55	49	52

Abbreviations: CTD, connective tissue disease; DM‐PM, dermatomyositis–polymyositis; MCTD, Mixed connective tissue disease; Others, rheumatoid arthritis and vasculitis.

Finally, diagnostic performances of EliA™ and LIA were compared for samples positive for Ro52 or Ro60 (CTD+) and positive for SARD (IFF+, and IFF−). As shown in Table 7, 25–36% of the samples positive for Ro52 and 37–41% of the samples positive for Ro60 could not be detected by IIF only.

**TABLE 7:** Diagnostic performance of ANA plus ENA screenings

SARD and Ro52/Ro60 positive	EliA™ CTD		LIA	
	Ro52	Ro60	Ro52	Ro60
IIF+ &CTD+	66	54	51	51
IIF−& CTD+	24	22	13	19
Loss Rate with IIF	36%	41%	25%	37%

Abbreviations: CTD, EliA™ CTD Screen; IIF, indirect immunofluorescence.

## 4 DISCUSSION

This study included patients with suspicion of SARD referred from primary care, rheumatology, nephrology, and internal medicine departments. It is important to distinguish these different groups of patients which generally are referred to as SARD or ANA‐associated rheumatic diseases (AARD) as there is considerable variability among them.^9^, ^10^, ^11^ In our study cohort, the percentages of patients detected by IIF (55%, *n* = 175) were similar to those obtained in the routine work of our laboratory and in other studies such as Bizzaro et al.^5^ and Dellavance et al.^12^ which reported prevalence rates of 46.7% and 44.3%, respectively, which also used a 1/80 dilution as a cut‐off point as we used in this study.

The comparative analysis of the three screening methods revealed that the sensitivities and specificities of the IIF and ELISA methods were more similar to each other (69% sensitivity in both methods, and 56% and 74% specificity in IIF and ELISA, respectively), while EliA™ CTD Screen had higher sensitivity and specificity levels for SARD (79% sensitivity, 91% specificity). Positive and negative LRs (8.33 and 0.23, respectively) were also better than IIF and ELISA. The sensitivity and specificity values for IIF were lower than reported by Orme et al.^2^ at 1/80 titer (sensitivity between 84% and 93%, and specificity between 62% and 81%) in a meta‐analysis performed to compare the diagnostic accuracy of IIF and EliA™ CTD Screen methods. However, in the same study, sensitivity and specificity values for EliA™ CTD Screen (sensitivity between 71% and 84%, and specificity between 90% and 96%) were similar to the values demonstrated in this publication. This has been also reported by Bizzaro et al.^13^ In their study, the diagnostic efficiency of EliA™ CTD Screen and IIF obtained by seven different studies was compared, obtaining an average efficiency of 87.1% for EliA™ CTD Screen and 77.1% for IIF (ranges between 77%‐96% and 70–87%, respectively). The performance obtained in the present study for EliA™ CTD Screen is consistent with the numbers reported by Bizzaro; while for IIF, we have obtained slightly lower values. It is important to mention that the results of IIF and ELISA methods have been shown to be highly dependent on the equipment used and also between equipment from the same manufacturer.^14^ In a study that evaluated the sensitivity of automated IIF and ELISA, the authors reported values between different manufacturers ranging from 77.7% and 95.5% for IIF, and Immunoconcepts HEp‐2 cells were the ones displaying the lowest value (77.7%). Values were higher for ELISA, 88.3% and 86.4% for the two studied methods.^15^ It should also be noted that the performance of IIF may vary among laboratories, and it is more consistent in fully automated tests.^16^

When the diagnostic efficiency of combined tests was evaluated (EliA™ CTD Screen plus IIF, and ELISA plus IIF), sensitivity and negative LR values were observed to be increased compared to EliA™ CTD Screen or ELISA alone measurements. In particular, EliA™ CTD Screen plus IIF combination results were highly promising with high sensitivity and low negative LR (sensitivity = 93% and negative LR = 0.14). Although combination tests had lower specificity and positive LR values compared to individual tests, sensitivity was higher and negative LR was below the limits considered clinically significant.^4^ These results are in line with published studies and current recommendations which advocate for the joint performances of IIF and a screening test with a large number of solid‐phase fixed antigens, such as the EliA™ CTD Screen (FEIA), or chemiluminescence assays.^5^, ^16^ Second‐generation ANA screening approaches have been developed combining IIF with antigenic specificity assays in a single test consisting of HEp‐2 cells immobilized in a central compartment of a glass holder and surrounded by microparticles with specific antigens.^17^

Higher measuring agreement has been observed between EliA™ CTD Screen and ELISA compared to EliA™ CTD Screen and IIF, most likely due to the fact that EliA includes all the antigens with enhanced expression in ELISA. In EliA™ CTD Screen a mixture of 17 ANAs associated with SARD was present, while in ELISA there were 12. On the other hand, IIF uses a much wider range of antigens. As assessed by Pearson's chi‐square test, although there were statistically significant differences between the results of the methods (alone or in combination), when EliA™ CTD Screen or ELISA were combined with IIF, sensitivity, specificity, and measure agreement improved. The meta‐analysis by Orme et al.^2^ has also reported significant differences between the diagnostic odds ratios of EliA™ CTD Screen and IIF.

For EliA™ CTD Screen positive samples, specific antibodies associated with SARD have been tested, using LIA and EliA™ (FEIA). However, dsDNA has not been considered for the study, since it would not be used for comparing LIA and FEIA, as it is not recommended to analyze anti‐dsDNA autoantibodies by LIA.^18^ One or more antibodies were detected by LIA in 84% of SARD samples in our population, while in 89% of the samples antibodies were detected by FEIA. It is remarkable that, in the 18 EliA™‐CTD positive SARD negative samples, 43% and 38% were positive in LIA and FEIA tests, respectively; among these, anti‐SSA/Ro (60 kD) autoantibodies were detected in 82% of the cases and anti‐centromere autoantibodies in 18%. Anti‐SSA/Ro (60 kD) antibodies have been reported to be among the ANA most frequently detected in routine SARD testing.^19^ They are mainly detected in patients with systemic lupus erythematosus and Sjögren's disease.^20^ In our cohort, anti‐Ro60 autoantibodies were more prevalent than anti‐Ro52. Interestingly, up to 41% of the samples positive for Ro60 could not be detected when IIF was used as the only screening method.

The study has three main limitations. The first is sample size. The cohort size (*n* = 323) was limited; however, it can be considered representative of the routine work of a laboratory specialized in the diagnosis of SARD. The second relies on the fact that the analysis of antibodies against the different antigenic specificities associated with SARD was performed exclusively in patients with positive EliA™ CTD Screen following the usual protocol of the laboratory. Finally, neither disease duration nor concurrent therapies and disease activity were considered for the analysis, so its impact on the results has not been evaluated.

In summary, EliA™ CTD Screen presents better diagnostic performance results versus IIF and ELISA individual measurements. The combined use of IIF with a screening test based on the use of solid‐phase fixed antigens, such as EliA™ CTD Screen, clearly improves the detection capacity of SARD (sensitivity = 93% and LR− = 0.14). Furthermore, the use of EliA™ CTD Screen as first‐line screening technique allows the detection of Ro52 and Ro60 antibodies (considered two of the most prevalent antibodies in SARD) which would not be detected by IIF alone, thus increasing the false‐negative rate.

## CONFLICT OF INTEREST

Dr. González has received a speaker honorarium from Thermo Fisher Scientific.

## AUTHOR CONTRIBUTIONS

All authors confirmed they have contributed to the intellectual content of this paper and have met the following 3 requirements: (a) significant contributions to the conception and design, acquisition of data, or analysis and interpretation of data; (b) drafting or revising the article for intellectual content; and (c) final approval of the published article.

## ETHICS APPROVAL

This study was approved by the Ethics Committee of the University of Virgen Macarena Hospital (Approval reference number: 201932115312). Authors declare that the manuscript has not been submitted to any other journal for simultaneous consideration, has not been published previously and data have not been manipulated or copied from other authors.

## Data Availability

The datasets used and/or analyzed during the current study are available from the corresponding author on reasonable request.
